# Developing a tool to measure tuberculosis-related stigma in workplaces in Indonesia: An internal validation study

**DOI:** 10.1016/j.ssmph.2023.101337

**Published:** 2023-01-10

**Authors:** Dewi Sumaryani Soemarko, Frisca Aprillia Halim, Aria Kekalih, Faisal Yunus, Retno Asti Werdhani, Agus Sugiharto, Muchtaruddin Mansyur, Tom Wingfield, Ahmad Fuady

**Affiliations:** a Department of Community Medicine, Faculty of Medicine, Universitas Indonesia, Jl. Pegangsaan Timur No.16, Jakarta, 10310, Indonesia; b Master of Occupational Medicine Study Program, Faculty of Medicine, Universitas Indonesia, Jl. Pegangsaan Timur No.16, Jakarta, 10310, Indonesia; c Department of Pulmonology and Respiration, Faculty of Medicine, Universitas Indonesia, Jl. Persahabatan Raya No.1, Jakarta, 13230, Indonesia; d South East Asian Ministers of Education Organization Regional Center for Food and Nutrition, Jakarta, 13120, Indonesia; e Departments of Clinical Sciences and International Public Health, Liverpool School of Tropical Medicine, Liverpool, L3 5QA, UK; f Department of Global Public Health, WHO Collaborating Centre on Tuberculosis and Social Medicine, Karolinska Institute, Stockholm, 171 76, Sweden; g Tropical and Infectious Disease Unit, Royal Liverpool and Broadgreen University Hospitals NHS Trust, Liverpool, L7 8XP, UK; h Primary Health Care Research and Innovation Center, Indonesian Medical Education and Research Institute, Faculty of Medicine Universitas Indonesia, Jl. Salemba Raya No.6, Jakarta, 10430, Indonesia

**Keywords:** Tuberculosis, Stigma, Worker, Tool, Validation, Indonesia, TB, Tuberculosis, WHO, World Health Organization, CFA, Confirmatory Factor Analysis, EFA, Exploratory Factor Analysis, ISPOR, International Society for Pharmacoeconomics and Outcomes Research

## Abstract

Workers with tuberculosis (TB) are often stigmatized, negatively impacting their socioeconomic position, mental health, and TB treatment outcomes. There is a dearth of validated tools to assess stigma in the worker population. This study aimed to develop and validate a novel, culturally adapted tool to measure TB-related stigma among workers in Indonesia. We translated, adapted, applied, and internally validated Van Rie's TB-Stigma Scale to the worker population in varying sizes businesses (formal and informal business sectors) in Indonesia. Psychometric evaluation using exploratory and confirmatory factor analyses (EFA and CFA) was performed to check the tool's internal consistency and reliability. The translation and cultural adaptation phases resulted in a final 11-item tool. From 172 participant responses, the EFA found two loading factors relating to responses on isolation and exclusion from the workplace. The CFA confirmed that the developed model had moderate fit with R^2^ values for each item ranging from 0.37 to 0.84. The tool was reliable (Cronbach's alpha 0.869). This validated, consistent and reliable adapted tool is ready to use in larger scale evaluations of TB-related stigma amongst workers in formal and informal business sectors of Indonesia to develop strategies to eliminate TB-related stigma from the workplace.

## Highlights

• This is the first tool to measure stigma towards people with tuberculosis, developed for the general worker population.
• The tool was adapted from Van Rie's TB Stigma Scale with adjustments and additional items relevant to the worker population.
• The tool is valid, consistent, reliable, and ready to use in larger-scale evaluations of TB-related stigma amongst workers.

## 1 Introduction

Tuberculosis (TB) remains a substantial health problem in Indonesia, with an estimated 845,000 TB cases and 92,000 deaths in 2019 (World Health Organization, 2021). These figures, together with the high TB incidence in India, contributed to the significant global increase in TB notifications between 2013 and 2019 (World Health Organization, 2021). The Indonesian government has committed to reducing TB incidence and mortality by 2030, in line with the World Health Organization’s 2015 End TB Strategies targets (World Health Organization, 2015). However, efforts to achieve these targets are still hindered by significant challenges, including TB-stigma (Macintyre et al., 2017; Sommerland et al., 2017).

TB-stigma can negatively affect people's access to TB services, delay treatment, worsen treatment outcomes, and increase the likelihood of TB transmission within families and communities (Datiko et al., 2020). In addition, TB-stigma can also impact upon people's work life. About 30% of people with TB in Indonesia who have income-earning jobs, either in the formal or informal sector, lose their job because of their illness (Fuady et al., 2018). One of the drivers of job and income loss is stigma and discrimination against people with TB in the workplace. Such stigma can include but is not limited to TB-affected people having limited opportunities to get promotions, being isolated in the workplace, or being dismissed because they are perceived to have higher absenteeism and to be less productive, inefficient, or even burdensome to the enterprise's finances (International Labor Organization, 2018; World Health Organization, 2003). As a consequence, people with TB who are stigmatized in their workplace are at risk of facing financial and mental problems, such as anxiety and depression (Stop TB Partnership, 2019). Therefore, assessing stigma towards workers with TB is essential to understand TB-related stigma prevalence and determinants in the workplace and develop stigma-reduction strategies, policies, and legislation.

There have been several tools to assess TB-stigma (Stop TB Partnership, 2019), but most are those applied to people with TB, the general population, and healthcare workers who care for people with TB (Nuttall et al., 2022). Van Rie's TB-Stigma scale is one of the most used and adapted tools to assess TB-related stigma (Bergman et al., 2021; Van Rie et al., 2008). (See Appendix – Table S1) The Van Rie scale has two forms covering both community and patient perspectives toward TB, which address TB-Stigma comprehensively. The items in the scale can capture the types of stigma: (a) enacted or experienced stigma—the range of behaviours directly experienced by people with TB, (b) anticipated stigma—fear of negative behaviour of others towards people, and (c) internalised (or self) stigma—acceptance of negative stereotypes about people with TB. Identifying these types of stigma guides to finding key causes of stigma, such as fear of transmission, keeping distance from affected individuals, and moral values of blame, responsibility, guilt, and punishment (Stop TB Partnership, 2019; Van Rie et al., 2008).

To date, there has been no specific tool used to measure stigma towards fellow coworkers in workplaces and, according to our previous review, no intervention developed to address TB-stigma in this specific population (Nuttall et al., 2022). TB-Stigma in the workplace can develop in different ways from the stigma development in the general community, even in healthcare workers' settings. They work in more intense working hours, often in indoor settings, leading to increased fear of infection, stigmatization, and discrimination. In addition to the fear of transmission, the stigma in the workplace may develop from a productivity loss perspective. Some questions asked to the general population, for example, “Some people do not want those with TB playing with their children”, are irrelevant for workers and need adjustment. For these reasons, this study aimed to cross-culturally adapt Van Rie's TB-Stigma Scale in order to develop and validate a new tool to measure TB-stigma among workers in Indonesia. This study was a part of our larger work in developing a tool to measure TB-stigma and the psychosocial impact of TB among people with TB and their households in Indonesia (Fuady et al., 2022).

## 2 Materials and methods

**Fig. 1:**
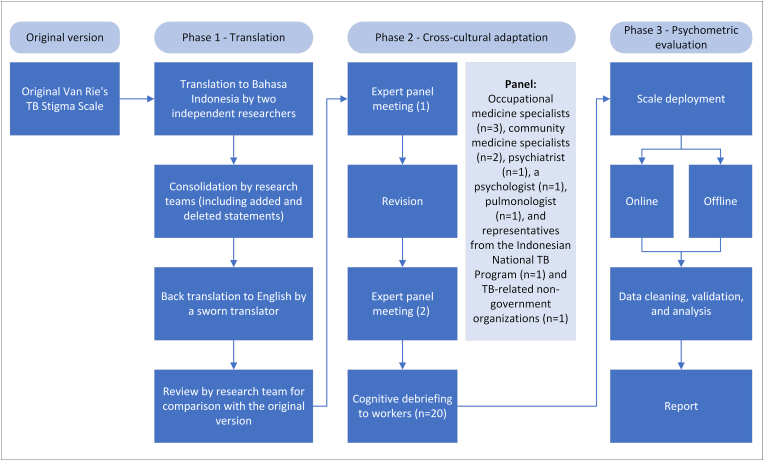
Flow of adaptation and validation process of the tool.

We performed the study in three phases between February and July 2022: Phase 1—Translation, Phase 2—Cross-cultural adaptation, and Phase 3—Psychometric evaluation. We conducted Phase 1 in two weeks, followed by Phase 2 in three months and Phase 3 in two months (one-month of data collection and one-month of data analysis) (Fig. 1).

### 2.1 Instrument

There is no tool, questionnaire, or scale to measure TB-stigma among workers in non-healthcare settings. We decided to adapt Van Rie's Stigma Scale which originally consisted of two parts: Part A: Community Perspectives towards TB (11 items); and Part B: Patient Perspectives towards TB (12 items). We used Part A for adaptation and validation in the general worker population on the assumption that in settings outside of healthcare this population would be similar to community respondents. Each of the 11 items in Part A of the Van Rie Stigma Scale has four options: strongly disagree (0), disagree (1), agree (2), and strongly agree (3) (Van Rie et al., 2008).

### 2.2 Phase 1: translation

The original Van Rie's TB-Stigma scale was translated into Bahasa, the lingua franca of Indonesia. Two independent researchers (FG, TS) fluent in English and Bahasa with previous experience in TB research did the translation separately, resulting in two versions of translated scales. The study team reconciled the two translated versions into one version, which was consolidated before the back translation. The consolidated version was then translated back to English by a contracted translator who was separate from the project team and did not know the original version of the tool. The adapted, back-translated English tool was subsequently reviewed and compared with the original tool by the study team to check for readability and consistency.

### 2.3 Phase 2: cross-cultural adaptation

We adapted the tool to the Indonesian context by (a) inviting local experts to a two-stage panel meeting and (b) pre-testing the tool prior to deployment. We purposively selected and invited thirteen experts with diverse but complementary experience: three occupational medicine specialists, three community medicine specialists, a psychiatrist, a psychologist, two pulmonologists, the Indonesian National TB Program officer, and two TB-related non-government organizations staff. Ten experts joined the first stage panel meeting. In this meeting, we invited suggestions from participants on the content and language of the tool in order to be culturally appropriate to the Indonesian context. Since there was no previous tool specific to the worker population, experts were invited to suggest additional items or delete items (resulting a Pre-final Tool 1). We invited the same experts to the second panel meeting. In this meeting, six experts provided further comments or suggestions to the Pre-final Tool 1 to shape the tool (Pre-final Tool 2). All panel expert meetings were recorded, and experts verbally consented at the beginning of the meetings.

We subsequently further revised the tool and sent to the panel experts to reach a version of the tool ready for pilot implementation (resulting in a Pilot Tool). We then did a cognitive debriefing by pre-testing the tool with 20 respondents in three enterprises in formal sector and one enterprise in informal sector, following the International Society for Pharmacoeconomics and Outcomes Research (ISPOR) guidance (Wild et al., 2005). The four enterprises were the enterprises included in this study. For the debriefing, we selected workers from different divisions of groups than those included in Phase 3. We deployed the tools in two forms but identical contents: online (developed in the REDCap platform) and paper-based, both of which were self-administered. We asked the respondents to check whether the statement items were straightforward, unambiguous, and not misinterpreted. We also asked respondents’ opinions on the content and language used in the tool and its appropriateness to the Indonesian cultural context. The study team discussed all inputs from the cognitive debriefing, did proofreading and finalized the tool for the psychometric evaluation.

### 2.4 Phase 3: psychometric evaluation

#### 2.4.1 Participant selection and sample size

For a psychometric evaluation, we first purposefully selected several enterprises in four provinces: East Java, West Java, Banten and Jakarta. We divided the enterprises into formal (medium to large size, more than 50 employees, and formally registered with the Ministry of Investment/Indonesian Investment Coordinating Board) and informal (small size, less than 50 employees, and not formally registered with the Ministry of Investment/Indonesian Investment Coordinating Board) business sectors. The enterprises in the formal sector were manufacturing, wholesale and service enterprises. The small enterprises in the informal sector were the home industry of bag makers, bakers, home chip makers, and local farmers. At each enterprise, we contacted the human resource department (formal sector) and the owner (informal sector) to select division(s) or group(s) of workers aged ≥18 years old. We calculated the sample size based on the original study's Cronbach's Alpha of 0.9 (Fuady et al., 2022) and assumed a Cronbach's Alpha of 0.85 in this study. With an alpha of 5% and power of 80%, we required at least 146 respondents (Bujang et al., 2018).

We also asked demographic characteristics of respondents, including sex, marital status, education level, workplace setting (indoor or outdoor), job level (high, middle, low), and their monthly income according to the Indonesian National Statistics Bureau's (grouped to three: < IDR 3.5 million [USD239], IDR 3–7.5 million [USD240-477] and > IDR 7.5 million [USD478]).

#### 2.4.2 Data collection and statistical analyses

We developed self-administered paper-based and online tools to allow flexibility in data collection. The online tool was developed using REDCap (https://redcap.fk.ui.ac.id) by FAH and AF. All tools were provided with a complete written explanation about the study and informed consent. Investigator (FAH) answered, clarified, and explained any questions from respondents regarding the tool.

We deployed an online-based tool to all workers in selected divisions/groups appointed by enterprise managers. The response rate was evaluated every two days, and if no response had been received, a same-day online message (i.e., WhatsApp messenger) was sent to remind invitees to fill out the tool. After seven days, we ended the data collection, assuming that we would not receive responses following three consecutive reminders. For enterprises in which online-based data collection were not possible, we deployed paper-based tool to the workers. All responses from the paper-based tool were entered into the REDCap platform (REDCap). All data were exported to IBM SPSS version 26 for data cleaning, validation, and analyses (IBM Corp, 2019).

#### 2.4.3 Internal consistency

We performed Exploratory Factor Analysis (EFA) to assess the internal consistency of the tool. In the Principal Axis Factor analysis, we set a threshold of 0.7 for Kaiser-Meyer-Olkin's (KMO) and 0.05 for Bartlett's test values. We followed the analyses with factor analysis by assessing the Eigenvalues to determine the number of factors or domains. We included factors with Eigenvalues >1 and contained three or more items with a loading of ≥0.4 (Bujang et al., 2018).

We applied a confirmatory factor analysis (CFA) and evaluated the model by calculating Root Mean Square Error of Approximation (RSMEA), Standardized Root Mean Square Residual (SRMR), Comparative Fit Index (CFI) and Tucker-Lewis Index (TLI). RSMEA value of less than 0.05 is considered as fit, while the value of 0.05–0.08 is a reasonable fit. CFI and TFI of more than 0.90 and SRMR of less than 0.08 were set as thresholds of model fitness. We also tested the reliability by assessing Cronbach's Alpha, with a Cronbach's Alpha coefficient of 0.80–0.90 being considered reliable (Bujang et al., 2018). CFA was done using the lavaan package in R.

### 2.5 Ethics

This study received ethical approval from the Research Ethics Committee of the Faculty of Medicine, Universitas Indonesia – Cipto Mangunkusumo Hosital, Jakarta, Indonesia (KET-60/UN2.F1/ETIK/PPM.00.02/2022. We provided information about the study to all respondents before they provided a consent to join the study, either by clicking “Agree” in electronic tool or signing in paper-based tool.

### 2.6 Reporting

Throughout the delivery and reporting of the study, we followed the guidance developed by ISPOR [17].

## 3 Results

### 3.1 Phase 1: translation

The study team proposed four main changes in translating and consolidating the tool. First, changing the subject in all statements, from "some people" to "I" because of the first-person perspective. In the original tool, people with TB were asked how they perceive the community's attitude toward them or people with TB. In this study, we asked workers about their attitudes toward people with TB so that using "I" is more relevant, contextual, and understandable. Second, we proposed the additional wording "coworkers" to replace "friends". These two changes reflected different perspectives from the original version—for example, from "Some people may not want to eat or drink with friends who have TB" to "I do not want to eat or drink with coworkers who have TB." Third, we proposed to delete one item, “Some people may not want to eat or drink with relatives who have TB” because it was not relevant for workers in their workplaces. Fourth, we proposed two additional items to explore whether people with TB were stigmatized in the workplace due to the perception that they were detrimental to their enterprise (see Appendix, Table S2).

1. I think that coworkers with TB have limited capacity to work *(Saya berpikir bahwa rekan kerja lain yang mengalami tuberkulosis memiliki kinerja yang terbatas)*
2. I think that coworkers with TB negatively impact the enterprise's finances *(Saya berpikir bahwa rekan kerja lain yang mengalami tuberkulosis merugikan perusahaan)*

These four changes were discussed at the expert panel meetings.

### 3.2 Phase 2: cross-cultural adaptation

All proposed changes were agreed upon in the first stage of the expert panel meeting (February 2022). In addition, one item, “I keep my distance from coworkers with TB” (adapted from the original item of ‘Some people keep their distance from people with TB'), was deleted because this item was represented by other items about keeping distancing, for example, ‘do not want to eat or drink with coworkers with TB’, 'feel uncomfortable around coworkers with TB’, and ‘behave differently around coworkers with TB’. This first stage panel meeting resulted in 11 items (Pre-final Tool 1). In the second stage (May 2022), there were no additional or deleted items, but some suggestions on the use of specific words, for example, changing ‘disgusting’ to ‘shameful’ and ‘afraid' to ‘worry’ for better understanding (See Appendix, Table S1).

In a cognitive debriefing, we deployed the Pilot Tool with 20 respondents representing those working in formal and informal sectors. All respondents filled in all items in the tool. When asked about the clarity of the tool, all respondents reported that the items were clearly understood. However, they suggested a few wording changes to improve the tool's clarity (See Appendix, Table S1). For the online-based tool, no technical issues (e.g., items not displayed correctly, missing items, unable to click the answer) were identified. There was no difference in interpretation between those filling out the online and the paper-based tool. No further substantial changes were made to the tool after this stage.

### 3.3 Phase 3: psychometric evaluation

We received 242 responses from the online tool and 96 responses from the paper-based tool. Of 242 online responses, 137 (56.6%) decided not to join the study, and 29 (11.9%) did not complete the tool. All responses from the paper-based tool were complete. Therefore, we entered 172 responses into the analysis.

Of 172 respondents, most were female (56%), married (68%), low-level staff (55%), worked in formal sectors (78%) and indoor settings (76%), and had low to moderate monthly income (<IDR3.5million [USD239], 52.3%) (Table 1). Most of the respondents (78%) were aware of TB. Thirty respondents had previously known coworkers who had been diagnosed with TB. Thirty-four respondents recognized that at least one person, either their coworker or someone outside their workplace, was rejected from their community or workplace due to being diagnosed with TB.

**Table 1:** Respondents’ characteristics (n = 172).

Characteristics	n	%
Sex		
Male	72	41.9
Female	100	58.1
Marriage status		
Single	39	22.7
Married	119	69.2
Widowed	14	8.1
Education level		
No schooling	3	1.7
Primary schooling	63	36.6
High school	66	38.4
College/University	40	23.3
Business sectors		
Formal sector	131	98.7
Informal sector	41	1.3
Workplace setting		
Indoor	137	79.7
Outdoor	35	20.3
Job level		
High (Director, Manager)	12	7.0
Middle (Supervisor)	22	12.7
Low (Staff, operator)	98	57.0
N/A (informal sector)	40	23.3
Monthly income		
< IDR3.5million (USD239)	90	52.3
IDR3.5–7.5m (USD240-477)	55	32.0
> IDR7.5m (USD478)	22	12.8
Prefer not to say	5	2.9
Previously diagnosed with TB		
Yes	6	3.5
No	166	96.5
Experience of coworkers or other people with TB		
Aware of TB	134	77.9
Has known coworkers with TB	30	17.4
Recognized people with TB (either coworkers or people outside workplace) who were rejected from workplace/community	34	19.7

### 3.4 Internal consistency

EFA of the 11-item adapted stigma tool gave a KMO value of 0.871 and a Bartlett's test value of 967.295 (*p*<0.001). Following on from these findings, further analysis identified two loading factors: isolation (V1, 2, 3, 4, and 5) and exclusion from the workplace (V6, 7, 8, 9, 10, and 11) (Table 2). The tool was reliable, with a Cronbach's alpha of 0.869.

**Table 2:** Loading factors of each tool item.

Items	Factor		Mean	Cronbach's Alpha if Item Deleted
	1	2		
V1 I do not want to eat or drink with coworkers with TB	0.553		1.92	0.867
V2 I feel uncomfortable about being near coworkers with TB	0.766		1.79	0.847
V3 I do not want to talk to coworkers with TB	0.610		1.42	0.859
V4 I try not to touch coworkers with TB	0.688		1.62	0.852
V5 I am worried about being infected by a coworker with TB	0.666		1.98	0.854
V6 I would behave differently towards coworkers with TB		0.464	1.15	0.861
V7 I do not want someone with TB working in my department/division/working room	0.510	0.538	1.61	0.849
V8 I think that a coworker with TB should be ashamed	0.422	0.466	1.31	0.857
V9 I think that a coworker with TB should be fired from his/her position		0.469	1.03	0.865
V10 I think that a coworker with TB has a more limited capacity to work than a coworker without TB		0.71	1.44	0.865
V11 I think that coworkers with TB can negatively impact the enterprise/workplace		0.72	1.17	0.859
Overall Cronbach's Alpha				0.869

**Fig. 2:**
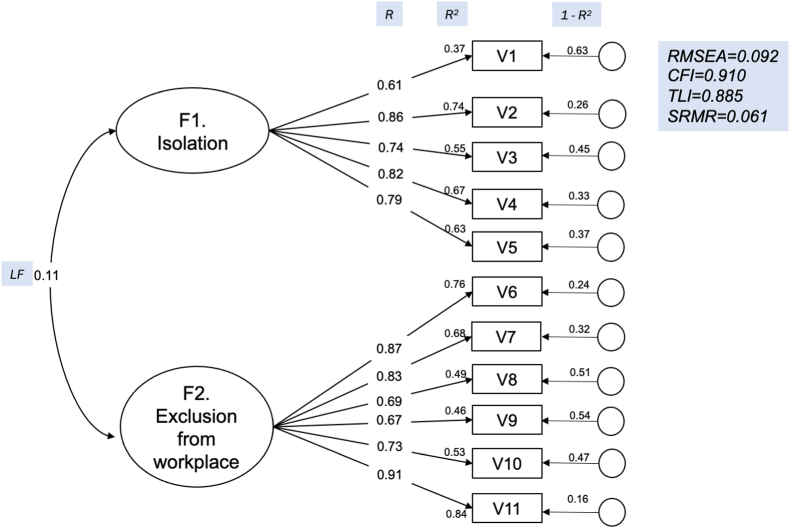
Confirmatory Factor Analysis of the tool. F: loading factors; V: tool's item; GFI: goodness-of-fit index; AGFI: adjusted GFI; RMSEA: root mean square error of approximation; NNFI (TLI): non-normed fit index (Tucker Lewis index); CFI: comparative fit index; LF: covariance between factors; R: variance indicating magnitude of relationship of items to factor; R^2^: percentage of variance of each item explained by factor; 1-R^2^: percentage of variance of each item not explained by factor.

In the CFA, we found that the scaled (robust) chi-square for our model was *X*^*2*^*(pdf)* = 105.58 (43), which was statistically significant (p<0.05, Fig. 2). The RMSEA value was 0.092, indicating that the model was reasonably fit, and we continued to further analysis. The SRMR was 0.061, with CFI of 0.910 and TLI of 0.885, showing the model was a reasonable fit. The R^2^ values for each item ranged from 0.37 to 0.84.

## 4 Discussion

We culturally adapted Van Rie's TB-Stigma Scale to develop a new TB-stigma tool to be applied to the worker population in Indonesia. The tool was considered comprehensive, had good content validity and internal consistency, and included some adapted and additional items relevant to the worker population.

This is the first tool developed to measure TB-Stigma among the non-healthcare worker population. This suggests that, despite people with TB anecdotally reporting stigmatization and discrimination in the workplace (Islam et al., 2015; Thu et al., 2012), such stigma is rarely measured and related legislation to protect workers with TB or symptoms of TB remains a neglected area (Stop TB Partnership, 2019). Two previous studies developed tools to measure TB-related stigma specific to healthcare workers in other countries (Sommerland et al., 2020; Wu et al., 2009). Some items used in these tools are similar to those used in the newly adapted tool. For example, "I do not want to work together with coworkers who have tuberculosis", "I am afraid of coworkers with tuberculosis", and "I do not want to eat or drink in the same room as a coworker who has tuberculosis” (Wouters et al., 2016, 2017) However, our tool was more comprehensive and was shown to have better consistency and validation.

Adaptation and validation of the tool followed the ISPOR's guidelines for adapting a tool to a new context. The experts involved in this study were medical specialists, psychologists, and professionals who work with people with TB, all of whom were well-informed on how stigma can develop in workplaces. Their involvement, alongside the participation of both formal and informal sector workers, also helped to refine the tool by formulating appropriate item wording from multiple diverse perspectives, adding, and deleting items to be more relevant to the context, and ensuring that every statement was understandable for the target population.

In adapting to a new context and population, we decided to ask the respondents items to consider in the first person “I” as opposed to third person "some people". Using the third person's perspective is generally applied to ask sensitive questions. In this context, besides improving the respondents' understanding of the statement, it was vital to capture their personal perceptions towards people with TB. We also replaced ‘people with TB’ with ‘coworkers with TB’ to highlight that the stigma being assessed was towards their coworkers.

The EFA showed two main factors running throughout the 11 items: isolation and exclusion from the workplace. These two factors are similar and may even intersect. However, isolation indicates perception and attitudes towards coworkers with TB who continue to work in the workplace. Conversely, exclusion from the workplace is a perception that coworkers with TB negatively impact the workplace and, therefore, should be excluded.

These two factors are helpful in identifying the roots of TB-Stigma in working place and how to tackle it. Isolation may be closely related to the misconception of TB development, spread, infection, and risk. Therefore, improving TB-related knowledge would significantly reduce TB-Stigma when the TB-related scores in this factor are high. Exclusion from the workplace may be related misconception of to the effects of TB. Workers with TB, especially those seeking care, may have impaired health, higher absenteism, and, therefore, may be assumed to be less productive or even costly for the enterprise. Enterprise managers may also not understand that people with pulmonary TB are generally non-infectious after 14 days of appropriate anti-TB therapy and therefore should be able to return to work after proper assessment. When the score is high for this ‘isolation’ factor, improving the perception of TB early detection, prompt treatment, evaluation, and enabling non-punitive sick leave is essential, particularly for those in a high-level position in the enterprise. Stronger legislation and social protection for those living with TB are also imperative to protect them from unnecessary job and income loss.

Two statements were added to this tool to capture exclusion from the workplace, “I think that coworkers with TB have limited capacity to work” and “I think that coworkers with TB negatively impact the enterprise's finances”. These statements showed relatively high loading factor values and were shown to be valid, internally consistent, reliable, and contributed to the overall internal consistency and reliability of the tool. These statements are also critical since TB-Stigma related to these statements can create health inequality for the stigmatized group. TB-Stigma, in this case, tends to keep people away (disease avoidance). In the workplace, stigmatization from the more powerful group (superintendent, manager, owner) to the less powerful group will create inequality in socioeconomic and health (Hatzenbuehler et al., 2013).

Despite the valuable findings, this study has several limitations. First, the respondents were those working on Java Island, which is characterised as a densely populated urban area with good access to information, including online information. The tool may not be generalizable to other areas including rural regions. Indonesia also has a wide variety of cultures that may affect the interpretation of question items and findings. Therefore, the items will need to be reviewed and refined prior to any wider implementation. Checking the wording and verifying the understanding of the statements among target respondents would help to optimize consistent and valid responses in future deployment. Second, the participation bias resulting from the high rejection rate among respondents receiving online tools may underestimate or overestimate the findings. Almost all respondents rejecting to join the study left the tool on its first page. We did not provide questions to explore the reasons for rejection, which can also be ethically problematic data to collect. Third, the proportion of respondents from the high-level position was low. Although we could assess the stigma related to exclusion from the workplace, it may not necessarily capture such stigma among males and those in a high-level position, who have more power to decide workers' employment status (Islam et al., 2015).

## 5 Conclusions

We successfully adapted Van Rie's TB-Stigma Scale into a tool to measure TB-Stigma amongst working people in Indonesia. The adapted tool is valid, internally consistent, reliable, and ready for wider external validation among workers in both formal and informal business sectors in Indonesia and beyond. Our identification of isolation and exclusion from the workplace as two significant loading factors may support the design and development of interventions, policies, and legislation to address the root causes of TB-Stigma in the workplace.

## Financial disclosure

This study had funding from PUTI Q1 Grant, 10.13039/501100006378Universitas Indonesia , Indonesia(Grant No. NKB-1104) and the 10.13039/501100000683Royal Society of Tropical Medicine and Hygiene, United Kingdom (Grant No. 19590206), received by Ahmad Fuady. Tom Wingfield is supported by grants from the 10.13039/100010269Wellcome Trust, UK (209075/Z/17/Z), the 10.13039/501100000265Medical Research Council, 10.13039/501100002992Department for International Development, and 10.13039/100010269Wellcome Trust, United Kingdom (Joint Global Health Trials, MR/V004832/1), and the 10.13039/501100009187Medical Research Foundation, United Kingdom (Dorothy Temple Cross International Collaboration Research Grant (MRF-131-0006-RG-KHOS-C0942).

The authors declare that they have no known competing financial interests or personal relationships that could have appeared to influence the work reported in this paper.

## Institutional review board statement

The Ethical Committee of Medicine, University of Indonesia approved this study with the ethical approval number KEP – 60/UN2·F1/ETIK/PPM.00.02/2022 and protocol number 22-01-0023.

## Informed consent statement

Informed consent was given to each respondent in this study. Respondents were also informed that they could resign or refuse if they were unwilling to participate in the study. In addition, they were informed that all information provided would be kept confidential.

## Author contributions

Conceptualization: FAH, DS, AF; Data curation: FAH, DS; Investigation: FAH, DS; Formal analysis: FAH, AF; Study management: FAH, AF, AS; Methodology: FAH, DS, AF, AK, MM, TW; Supervision: AF, AK, RAW, FY, AS, MM, TW; Validation: AF, AK, TW; Visualization: FAH, AF; Writing—original draft: FAH, DS, AF; Writing—review and editing: FAH, DS, AF, AK, RAW, FY, AS, MM, TW; Funding acquisition: AF, AS, TW.

## Data Availability

Data will be made available on request.
