# Supplementary material for: Developing a tool to measure tuberculosis-related stigma in workplaces in Indonesia: An internal validation study
Source: SSM Popul Health. 2023 Jan 10;21:101337. doi: 10.1016/j.ssmph.2023.101337 (PMC9842934; doi:10.1016/j.ssmph.2023.101337)
Supplement: Multimedia component 1 [file mmc1.docx]

**Appendix**

**Table S1**. Original TB-Stigma Scale by Van Rie *et al.* (2008)

| **Tuberculosis-related stigma scale items** | | | | |
| --- | --- | --- | --- | --- |
| **I. Community perspectives toward tuberculosis** |  |  |  |  |
| ***Instructions:*** *From now on, I shall read the statements, which explain about how your community feels towards people with TB. After I read each statement, please answer whether you agree or disagree that such events occur in your community. If you agree, I will ask how you agree, agree or strongly agree. If you disagree, I will ask how you disagree, disagree or strongly disagree. You can refuse to answer any questions that make you feel uncomfortable.* | | | | |
| **Items** | **Strongly Disagree** | **Disagree** | **Agree** | **Strongly Agree** |
| 1. Some people prefer not to have those with TB living in their community | □ | □ | □ | □ |
| 2. Some people keep their distance from people with TB | □ | □ | □ | □ |
| 3. Some people think that those with TB are disgusting | □ | □ | □ | □ |
| 4. Some people feel uncomfortable about being near those with TB | □ | □ | □ | □ |
| 5. Some people do not want those with TB playing with their children | □ | □ | □ | □ |
| 6. Some people do not want to talk to others with TB | □ | □ | □ | □ |
| 7. If a person has TB, some community members will behave differently towards that person for the rest of his/her life | □ | □ | □ | □ |
| 8. Some people may not want to eat or drink with friends who have TB | □ | □ | □ | □ |
| 9. Some people try not to touch others with TB | □ | □ | □ | □ |
| 10.Some people may not want to eat or drink with relatives who have TB | □ | □ | □ | □ |
| 11.Some people are afraid of those with TB | □ | □ | □ | □ |
| **II. Patient perspectives toward tuberculosis** |  |  |  |  |
| ***Instructions:*** *From now on, I shall read the statements, which explain about how people with TB feel. After I read each statement, please answer whether you agree or disagree that TB patients in your community feel like that. If you agree, I will ask how you agree, agree or strongly agree. If you disagree, I will ask how you disagree, disagree or strongly disagree. You can refuse to answer any questions that make you feel uncomfortable.* | | | | |
| **Items** | **Strongly**  **Disagree** | **Disagree** | **Agree** | **Strongly**  **Agree** |
| 1. Some people who have TB feel guilty because their family has the burden of caring for them | □ | □ | □ | □ |
| 2. Some people who have TB keep their distance from others to avoid spreading TB germs | □ | □ | □ | □ |
| 3. Some people who have TB feel alone | □ | □ | □ | □ |
| 4. Some people who have TB feel hurt of how others react to knowing they have TB | □ | □ | □ | □ |
| 5. Some people who have TB lose friends when they share with them they have TB | □ | □ | □ | □ |
| 6. Some people who have TB are worried about having AIDS | □ | □ | □ | □ |
| 7. Some people who have TB are afraid to tell those outside their family that they have TB | □ | □ | □ | □ |
| 8. Some people who have TB will choose carefully who they tell about having TB | □ | □ | □ | □ |
| 9. Some people who have TB are afraid of going to TB clinics because other people may see them there | □ | □ | □ | □ |
| 10.Some people who have TB are afraid to tell their family that they have TB | □ | □ | □ | □ |
| 11.Some people who have TB are afraid to tell others that they have TB because others may think that they also have AIDS | □ | □ | □ | □ |
| 12.Some people who have TB feel guilty for getting TB because of their smoking, drinking, or other careless behaviors | □ | □ | □ | □ |

**Table S2**.Transcultural Adaptation and Modification of the TB-Stigma Tool

| **No** | **Original Version** | **Tool Draft 1** | **Pre-final Tool 1** | **Pre-final Tool 2** | **Pilot Tool** |
| --- | --- | --- | --- | --- | --- |
| 1 | Some people may not want to eat or drink with friends who have TB | *Saya tidak ingin makan atau minum dengan rekan kerja yang mengalami tuberkulosis* | *Saya tidak* ***mau*** *makan atau minum bersama dengan rekan kerja lain yang mengalami tuberkulosis* ***/ TB / TBC*** | *Saya tidak* ***berkenan*** *makan atau minum bersama dengan rekan kerja lain yang mengalami tuberkulosis / TB / TBC* | *Saya tidak berkenan makan atau minum bersama dengan rekan kerja lain yang mengalami tuberkulosis / TB / TBC* |
| 2 | Some people feel uncomfortable about being near those with TB | *Saya merasa tidak nyaman berdekatan dengan rekan kerja yang mengalami tuberkulosis* | *Saya merasa tidak nyaman berdekatan dengan rekan kerja lain yang mengalami tuberkulosis* ***/ TB / TBC*** | *Saya merasa tidak nyaman berdekatan dengan rekan kerja lain yang mengalami tuberkulosis / TB / TBC* | *Saya merasa tidak nyaman berdekatan dengan rekan kerja lain yang mengalami tuberkulosis / TB / TBC* |
| 3 | If a person has TB, some community members will behave differently towards that person for the rest of his ⁄ her life | *Jika rekan kerja di tempat kerja saya mengalami tuberkulosis, saya akan berperilaku berbeda terhadapnya di tempat kerja dibandingkan sebelumnya* | *Jika rekan kerja di tempat kerja saya mengalami tuberkulosis* ***/ TB / TBC****, saya akan* ***bersikap*** *berbeda* ***terhadap orang tersebut*** *dibandingkan sebelumnya.* | ***Saya akan bersikap berbeda pada rekan kerja yang di diagnosis tuberkulosis/ TB/ TBC*** | *Saya akan bersikap berbeda pada rekan kerja yang di diagnosis tuberkulosis / TB / TBC* |
| 4 | Some people do not want those with TB playing with their children | *Saya tidak ingin seseorang yang mengalami tuberkulosis bekerja atau ditempatkan di departemen atau divisi saya* | *Saya* ***tidak mau*** *ada seseorang yang mengalami tuberkulosis* ***/ TB / TBC*** *bekerja atau ditempatkan di departemen atau divisi saya* | *Saya tidak mau ada seseorang yang mengalami tuberkulosis / TB / TBC bekerja atau ditempatkan di departemen atau divisi saya* | *Saya tidak mau ada seseorang yang mengalami tuberkulosis / TB / TBC bekerja atau ditempatkan di departemen / divisi /* ***ruang kerja*** *saya* |
| 5 | Some people keep their distance from people with TB | *Saya menjaga jarak dengan orang yang mengalami tuberkulosis di tempat kerja* | This statement is deleted because other statement has already captured any signs of distancing. | *-* | *-* |
| 6 | Some people think that those with TB are disgusting | *Saya berpikir bahwa rekan kerja yang mengalami tuberkulosis itu menjijikkan* | ***Menurut saya,*** *rekan kerja yang mengalami tuberkulosis* ***/ TB / TBC*** *itu menjijikkan* | *Menurut saya, rekan kerja yang mengalami tuberkulosis / TB / TBC itu* ***aib atau hal yang memalukan*** | *Menurut saya, rekan kerja yang mengalami tuberkulosis / TB / TBC itu aib atau hal yang memalukan* |
| 7 | Some people do not want to talk to others with TB | *Saya tidak ingin berbicara dengan rekan kerja yang mengalami tuberkulosis* | *Saya* ***tidak mau*** *berbicara dengan rekan kerja yang mengalami tuberkulosis* ***/ TB / TBC*** | *Saya tidak* ***berkenan*** *berbicara dengan rekan kerja yang mengalami tuberkulosis / TB / TBC* | *Saya tidak berkenan berbicara dengan rekan kerja yang mengalami tuberkulosis / TB / TBC* |
| 8 | Some people are afraid of those with TB | *Saya takut terhadap rekan kerja yang mengalami tuberkulosis* | *Saya* ***takut tertular*** *dengan rekan kerja lain yang mengalami tuberkulosis* ***/ TB / TBC*** | *Saya* ***khawatir*** *tertular dengan rekan kerja lain yang mengalami tuberkulosis / TB / TBC* | *Saya khawatir tertular dengan rekan kerja lain yang mengalami tuberkulosis / TB / TBC* |
| 9 | Some people try not to touch others with TB | *Saya berusaha tidak bersentuhan dengan rekan kerja yang mengalami tuberkulosis* | *Saya berusaha tidak bersentuhan dengan rekan kerja* ***lain*** *yang mengalami tuberkulosis* ***/ TB / TBC*** | *Saya berusaha tidak bersentuhan dengan rekan kerja lain yang mengalami tuberkulosis / TB / TBC* | *Saya berusaha tidak bersentuhan dengan rekan kerja lain yang mengalami tuberkulosis / TB / TBC* |
| 10 | Some people may not want to eat or drink with relatives who have TB | Not relevant in working place | *-* | *-* | *-* |
| 11 | Some people prefer not to have those with TB living in their community | *Saya berpikir bahwa rekan kerja yang mengalami tuberkulosis harus diberhentikan dari pekerjaannya* | ***Menurut saya,*** *rekan kerja lain yang mengalami* ***tuberkulosis / TB / TBC*** *harus dipecat dari pekerjaannya* | *Menurut saya, rekan kerja lain yang mengalami tuberkulosis / TB / TBC sebaiknya diberhentikan dari pekerjaannya* | *Menurut saya, rekan kerja lain yang mengalami tuberkulosis / TB / TBC sebaiknya diberhentikan dari pekerjaannya* |
| **Added items:** | | | | | |
| 12 |  | *Saya berpikir bahwa rekan kerja lain yang mengalami tuberkulosis memiliki kinerja yang terbatas* | *Saya berpikir bahwa rekan kerja lain yang mengalami tuberkulosis* ***/ TB / TBC akan*** *memiliki* ***kemampuan kerja*** *yang terbatas.* | *Saya* ***berpendapat*** *bahwa rekan kerja lain yang mengalami tuberkulosis / TB / TBC akan memiliki kemampuan kerja yang terbatas.* | *Saya berpendapat bahwa rekan kerja lain yang mengalami tuberkulosis / TB / TBC* ***pasti*** *akan memiliki kemampuan kerja yang terbatas.* |
| 13 |  | *Saya berpikir bahwa rekan kerja lain yang mengalami tuberkulosis merugikan perusahaan* | *Saya berpikir bahwa rekan kerja lain yang mengalami tuberkulosis* ***/ TB / TBC*** ***dapat*** *merugikan perusahaan* | *Saya* ***berpendapat*** *bahwa rekan kerja lain yang mengalami tuberkulosis / TB / TBC dapat merugikan perusahaan* | *Saya berpendapat bahwa rekan kerja lain yang mengalami tuberkulosis / TB / TBC dapat merugikan perusahaan /* ***tempat kerja*** |
